# Genome Sequence of the Tropical Atmosphere Bacterium *Pontibacter* sp. Strain SGAir0037

**DOI:** 10.1128/MRA.00610-19

**Published:** 2019-09-12

**Authors:** Serene B. Y. Lim, Ana Carolina M. Junqueira, Akira Uchida, Rikky W. Purbojati, James N. I. Houghton, Irvan Luhung, Caroline Chénard, Anthony Wong, Sandra Kolundžija, Megan E. Clare, Kavita K. Kushwaha, Alexander Putra, Nicolas E. Gaultier, Balakrishnan N. V. Premkrishnan, Cassie E. Heinle, Vineeth Kodengil Vettath, Daniela I. Drautz-Moses, Stephan C. Schuster

**Affiliations:** a Singapore Centre for Environmental Life Sciences Engineering, Nanyang Technological University, Singapore; b Departamento de Genética, Instituto de Biologia, Universidade Federal do Rio de Janeiro, Rio de Janeiro, Brazil; University of Maryland School of Medicine

## Abstract

The Pontibacter bacterial genus has been detected in marine and soil environments. Here, we report the genome sequence of *Pontibacter* sp. strain SGAir0037, which was isolated from outdoor air samples collected in Singapore. The genome comprises one chromosome of 5.26 Mb and one plasmid of 127 kb.

## ANNOUNCEMENT

Members of the genus Pontibacter have recently been added to the family *Hymenobacteraceae* (1), phylum *Bacteroidetes*. The bacterium Pontibacter actiniarum was the first species of the genus to be discovered and was initially classified within a group of unidentified marine actinians living at a depth of 100 m within the Rudnaya Bay, East Sea (2). Later studies also detected Pontibacter spp. in additional habitats, including solar salterns (3), drainage systems (4), desert soil (5), and even human blood culture (6).

*Pontibacter* sp. strain SGAir0037 is an orange-pigmented bacterium and presents as rod-shaped cells under scanning electron microscopy (SEM), which is similar to what is found with previously reported members within this genus (6). SGAir0037 was isolated from an aerosol sample collected in Singapore (1.346N, 103.680E). Air was drawn in and directly impacted onto a blood agar base (Oxoid, UK) mounted in an Andersen single-stage impactor (SKC, USA). After initial incubation at 25°C, subsequent isolation of pure colonies was carried out by culturing on Trypticase soy agar (Becton, Dickinson, USA) at 30°C. Finally, the pure culture was grown in lysogeny broth (Becton, Dickinson) overnight before DNA extraction.

Genomic DNA was extracted following the standard protocol of the Wizard genomic DNA purification kit (Promega, USA). Single-molecule real-time (SMRT) sequencing was subsequently performed on the PacBio RS II platform (Pacific Biosciences, USA) after libraries were constructed using the SMRTbell template prep kit 1.0 (Pacific Biosciences). In addition, whole-genome shotgun libraries were constructed with the TruSeq Nano DNA library preparation kit (Illumina, USA) and sequenced on the MiSeq platform (Illumina) with a 600-cycle paired-end sequencing run to generate short reads.

Default parameters were used for all software unless otherwise specified. Genome assembly was quality controlled using PreAssembler filter version 1 within the Hierarchical Genome Assembly Process version 3 (HGAP3) (7) for PacBio reads and using Cutadapt version 1.8.1 (8) for MiSeq reads. The PacBio RS II platform generated 113,495 subreads which were *de novo* assembled using HGAP3 that is part of the PacBio SMRT Analysis 2.3.0 package. The assembly was polished using Quiver (7), while assembly errors were corrected using Pilon version 1.16 (9) (–tracks –changes –vcf –fix all –mindepth 0.1 –mingap 10 –minmq 30 –minqual 20 –K 47) with the 939,311 MiSeq short reads.

The assembly of the *Pontibacter* sp. strain SGAir0037 genome generated two circular contigs, which were confirmed with Circlator version 1.1.4 (10), one being a chromosome of 5,256,878 bp (198-fold coverage) and one being a circular plasmid of 126,548 bp (136-fold coverage). The chromosomal contig has a G+C content of 45.2%, while the plasmid pSGAir0037 has 48.8% G+C content. Validation of plasmid pSGAir0037 was performed by BLASTn analysis against 13,789 recorded plasmids in the PLSDB database (11), resulting in 89.9% identity to NCBI RefSeq accession number NZ_CP021236 (Pontibacter actiniarum DSM 19842 plasmid, complete sequence).

Average nucleotide identity (ANI) was evaluated with Microbial Species Identifier (MiSI) version 1.0 (12), which was run using ANICalculator. The analysis was carried out against a database of 6,387 bacterial RefSeq genomes which was created using a text filter for “type, synonym type, proxytype” and the subsequent getorf -find 3 option. The top results showed only 74.9% similarity and 22% alignment fraction with *P. actiniarum* strain DSM 19842. A subsequent BLASTn analysis (BLASTN 2.9.0+) (13) against the nucleotide collection (nr/nt) database of the 16S rRNA gene sequence of *Pontibacter* sp. strain SGAir0037 showed 94.8% identity and 95.0% query coverage to the genus *Pontibacter*.

The assembled genome was also annotated using NCBI’s Prokaryotic Genome Annotation Pipeline (PGAP) version 4.2 (14). The annotation predicted 4,557 genes, with 4,457 protein-coding genes, 6 copies each of 5S, 16S, and 23S rRNAs, 44 tRNAs, 3 noncoding RNAs, and 35 pseudogenes.

Functional annotations with Rapid Annotations using Subsystems Technology (RAST version 2.0, with annotation scheme “Classic RAST,” added automatically fix errors, fix frameshifts, and backfill gaps [15–17]) indicated 16 genes associated with nitrogen metabolism. To date, Pontibacter diazotrophicus is the only species in the genus associated with nitrogen-fixing capability (18). Furthermore, 17 genes were identified as being associated with menaquinone biosynthesis, which is an essential component of the electron transfer pathway in prokaryotes (19). No motility-associated genes were detected, suggesting a passive form of transport for this bacterium.

### Data availability.

The complete genome sequences of *Pontibacter* sp. strain SGAir0037 and its plasmid pSGAir0037 have been deposited in DDBJ/EMBL/GenBank under the accession numbers CP028092 and CP028093, respectively. The accession numbers of the raw reads in the SRA database are SRR8894375, SRR8894376, and SRR8894377.
